# Study on the extraction and stability of total flavonoids from *Millettia speciosa* Champ

**DOI:** 10.1371/journal.pone.0326570

**Published:** 2025-07-02

**Authors:** Junhai Liu, Xiaosha Guo, Tengteng Yang, Xiaoli Wang, Yinku Liang

**Affiliations:** 1 Shaanxi Key Laboratory of Catalysis, Shaanxi University of Technology, Hanzhong, Shaanxi, China; 2 School of Chemistry and Environment Science, Shaanxi University of Technology, Hanzhong, Shaanxi, China; Museu Paraense Emilio Goeldi, BRAZIL

## Abstract

*Millettia speciosa* Champ, a member of the Leguminosae family and the genus *Millettia*, is a rich source of total flavonoids. Investigating the extraction methods and stability of total flavonoids from *Millettia speciosa* Champ using ultrasound-assisted extraction is crucial for enhancing its medicinal value and advancing the modernization of traditional Chinese medicine. In this study, the optimal extraction conditions were determined as follows, ethanol volume fraction of 64%, ultrasonic temperature of 60°C, solid-liquid ratio of 1:20 g/mL, ultrasonic time of 29 min, and ultrasonic power of 400 W. Under these conditions, the extract of total flavonoids from *Millettia speciosa* Champ was 6.485%. The stability of the total flavonoids was evaluated under various conditions. The results indicated that the total flavonoids should be stored in the dark. Metal ions such as K^+^, Na^+^, Ca^2+^, Mg^2+^, and Al^3+^ had no significant impact on stability, whereas Cu^2+^, Zn^2+^, and Fe^3+^ caused a notable loss of stability. The total flavonoids remained stable under neutral and weakly acidic conditions but were unstable under strongly acidic and alkaline conditions. Additives such as sodium benzoate, sodium chloride, glucose, and sucrose did not significantly affect stability. In contrast, ascorbic acid and citric acid had minimal effects on stability at low concentrations but significantly impacted stability at higher concentrations.

## 1. Introduction

*Millettia speciosa* Champ is a plant species belonging to the legume family (*Fabaceae*) and the genus *Millettia*. It is commonly found in hillside meadows and is primarily distributed in tropical and subtropical regions. This species thrives in warm and humid climates, favoring fertile soils with good drainage for optimal growth [1,2].

*Millettia speciosa* Champ smells sweet and fragrant, mild, has the effect of tonifying the deficiency and moistening the lungs, strengthening the tendons and activating the collaterals, aphrodisiacs, nourishing the kidneys and replenishing the deficiency, etc [3,4]. It is mainly used for the treatment of lumbar muscle strain, rheumatoid arthritis, paralyzing pain in the joints, stiffness of the joints and other symptoms, and has a good therapeutic effect on the respiratory diseases such as lung fever, coughing of the deficiency of the lungs, pulmonary tuberculosis, chronic bronchitis, chronic hepatitis, etc. and liver diseases [5–7]. Wang et al., [8] were comprehensively evaluated the nutritional value, chemical composition, biological activities, and feed indices of different parts of *Millettia speciosa* Champ. The results showed the extremely high nutritional value. The antibacterial activities of the flower and seed extracts were significantly stronger than those of the leaves and branches. The leaf extract displayed the strongest antifungal activities. In addition, the potential applicability of *Millettia speciosa* Champ as an animal feed [9]. Moreover, *Millettia speciosa* Champ vigorously also has a regulating effect on male spermatorrhea and female leucorrhea. Another aspect of *Millettia speciosa* Champ is also a common ingredient that is often eaten in soup with other ingredients such as pork bones, chicken and five-fingered peaches [10]. The root can be used as medicine after washing, slicing and drying, or steaming and drying, which is the main medicinal part of *Millettia speciosa* Champ [11,12].

With the in-depth research on the medicinal value of natural medicines and plant extracts, total flavonoids have attracted widespread attention for their significant antioxidant, anti-inflammatory, anti-tumour and other biological activities. In the past three decades alone, several isoflavonoids originating from *Millettia speciosa* Champ have been isolated, and their pharmacological activities have been evaluated against major diseases, such as cancer, inflammation, and diabetes [13]. Yu et al., [14] unequivocally identified 21 isoflavones and 4 isoflavones from the Roots of *Millettia speciosa* Champ by ethanol extraction followed by further extraction with chloroform. A total of 35 total flavonoids were identified by Wang et al., [12] in the extracts of *Millettia speciosa* Champ roots. The extracts reduced body weight gain, liver weight gain, white adipose tissue, lipid accumulation, and blood glucose. The levels of TG, ALT, AST, and inflammatory-related adipokines (TNF-α and IL-6) in serum were also reduced by the extracts. It was shown that long-term administration of these extracts may improve obesity by stimulating fat thermogenesis and lipid metabolism. Zhang et al., [11] studied the chemical profile and ameliorating effect on glycolipid metabolism of *Millettia speciosa* champ. It was found to be rich in total flavonoids and alkaloids, which might support the potential relation of material foundation and the activity in regulating glycolipid metabolism. The ameliorating effect on glycolipid disorder in diabetic mice might be associated to the regulation of related hormones of the HPA axis and the IRS2/PI3K/Akt/GLUT4 signalling pathway. It was of great significance for advanced directed separation and pharmacological activity research of MSC. As a traditional Chinese medicine, *Millettia speciosa* Champ is rich in total flavonoids and has a wide range of application prospects [6]. Therefore, the development of an efficient and stable method for the extraction of total flavonoids from *Millettia speciosa* Champ is of great significance for improving its medicinal value and promoting the modernization of traditional Chinese medicine.

In recent years, the research on the extraction technology of total flavonoids has made remarkable progress [15,16]. However, the traditional extraction methods, such as solvent extraction and reflux extraction, have some problems such as low extraction efficiency and complicated operation [17,18]. Ultrasonic-assisted extraction technology, as a new extraction method with the advantages of easy operation and high extraction efficiency, has been widely used in the extraction process of natural products [19–21]. Zhen et al., [22] compared Ultrasonic-assisted extraction (UAE), microwave-assisted extraction (MAE) and conventional soxhlet extraction (CSE), and the results showed that UAW is the best extraction method for extracting total flavonoids from *Millettia speciosa* Champ, and that total flavonoids of *Millettia speciosa* Champ had great antioxidant activities. At the same time, the stability of total flavonoids is also a key factor affecting their efficacy [23,24]. During extraction and preservation, total flavonoids are susceptible to degradation or transformation by environmental factors such as light, temperature, humidity, etc., leading to a decrease in efficacy [25–27]. Therefore, the study on the stability of *Millettia speciosa* Champ total flavonoids under different conditions is of great significance for ensuring the stability of its efficacy and extending its shelf life.

In this study, ultrasonic-assisted extraction technology was adopted to extract the total flavonoids from *Millettia speciosa* Champ, on the basis of single-factor experiments, the extraction factors were optimized through the response surface analysis methodology, including the volume fraction of ethanol, ultrasonic temperature, solid-liquid ratio, ultrasonic time, and ultrasonic power, so as to improve the extraction rate of total flavonoids. Meanwhile, the stability of total flavonoids obtained by extraction was investigated in light, temperature, pH, metal ions, and food additives. The aim of this study was to maximize the extraction rate of total flavonoids from *Millettia speciosa* Champ, as well as to explore the physicochemical properties of total flavonoids, which would further provide strong support for the development and utilization of total flavonoids from *Millettia speciosa* Champ.

## 2. Materials and methods

### 2.1. Plant material

*Millettia speciosa* Champ (naturally air-dried) originating from Lufeng County, Yunnan Province, was purchased from the Hanzhong pharmacy market. The stem parts of *Millettia speciosa* Champ were used for the extraction of total flavonoids.

The *Millettia speciosa* Champ was cleaned with distilled water, and dried until the quality was constant. The *Millettia speciosa* Champ seed powder was obtained by crushing the *Millettia speciosa* Champ with a 40-mesh sieve and put into a brown grinding glass bottle for use.

### 2.2. Chemicals

Rutin standard (HPLC≥98%) was purchased from Shanghai Chemical Reagent Company. Anhydrous ethanol (C_2_H_5_OH, 99%), potassium chloride (KCl, 98%), calcium chloride (CaCl_2_, 99.0%), magnesium chloride (MgCl_2_, ≥ 98%), cupric chloride (CuCl_2_, 99%), zinc chloride (ZnCl_2_, ≥ 98%), ferric chloride(FeCl_3_, 98%), aluminium chloride (AlCl_3_, 99%), sodium benzoate (C_7_H_5_NaO_2_, ≥ 99%) were purchased from Fuchen (Tianjin) Chemical Reagent Co., Ltd. Ascorbic acid (C_6_H_8_O_6_, > 99.0% (T)), citric acid (C_6_H_8_O_7_, 99.5%), glucose (C_6_H_12_O_6_, 98%), sucrose (C_12_H_22_O_11_), sodium nitrite (NaNO_2_, 99%) and sodium hydroxide (NaOH, 98%) were obtained from Aladdin Chemistry Co. Ltd. All the chemicals were used without any further purification. All studies were performed using 18.2 MΩ cm de-ionized water.

### 2.3. Main instruments

GR-200 Electronic Balance Shanghai Guangzheng Medical Instrument Co., Ltd. FW117 Chinese herbal medicine crusher Tianjin Tester Instrument Co., Ltd. GL1030 Ultrasonic Cleaner Shenzhen Guanbo Technology Industry Co., Ltd. DGG-9140B Electric Heat Constant Temperature Blower Dryer Shanghai Senxin Test Instrument Co., Ltd. HH-2 type electric heating constant temperature water bath pot Bohai Electric Appliance Factory, Huanghua City, Hebei Province. H1850 low speed centrifuge Beijing Jingli Centrifuge Co., Ltd. Cary5000 UV-Vis spectrophotometer Valian Medical System Company. SHZ-D9 (III) circulating water vacuum pump Gongyi Yuhua Instrument Co., Ltd.

### 2.4. Extraction of total flavonoids

*Millettia speciosa* Champ powder (1.0 g) was accurately weighed in a 50 mL dry beaker, and 30 mL ethanol aqueous solution with 50% volume fraction was added to the ultrasonic cleaner. The temperature was set at 40°C and the ultrasonic power was 80 W. Then ultrasonic extraction was carried out for 40 min. After ultrasonic extraction, the sample was transferred to a 10 mL centrifuge tube and centrifuged at 3000 r/min for 10 min. The extract was filtered and collected, and the volume (V) was measured.

### 2.5. Single factor experiments for the extraction of total flavonoids

Based on the extraction process of 2.4, single factor experiments were carried out by changing the ethanol volume fraction (40%, 50%, 60%, 70% and 80%), solid-liquid ratio (1:10, 1:15, 1:20, 1:25, and 1:30 (g/mL)), ultrasonic temperature (40, 50, 60, 70, and 80°C), ultrasonic power (200, 300, 400, 500, and 600 W) and ultrasonic time (10, 20, 30, 40, and 50 min) to explore the effects of these five factors on the extract of total flavonoids from *Millettia speciosa* Champ. The orthogonal experimental design table is shown in S1 Table.

### 2.6. Optimization of extraction conditions

Subsequently, a quadratic regression orthogonal experimental design was employed to develop a more precise mathematical model. This approach allowed for the quantification of nonlinear relationships among the factors and facilitated the optimization of experimental conditions. The specific details of the quadratic regression orthogonal experimental design are provided in S4 Table.

### 2.7. Determination of total flavonoids

1 mL of the prepared extract was added to a 100 mL volumetric flask, and then the following NaNO_2_, Al(NO_3_)_3_, NaOH aqueous solution, and 70% ethanol aqueous solution were added in sequence. The absorbance value of the spectrophotometer was controlled in the range of 0.2–0.8, and the dilution ratio (N) was recorded. With 70% ethanol aqueous solution, and NaNO_2_ and NaOH mixed aqueous solution as blank controls, the maximum absorption wavelength was determined by a UV-Vis spectrophotometer in the range of 400–800 nm. The results showed that the *Millettia speciosa* Champ extract had a maximum absorption peak at 510.0 nm, which was consistent with the maximum absorption wavelength of the rutin standard substance at 510.0 nm, indicating that the absorbance spectrum of the crude extract of *Millettia speciosa* Champ is consistent with rutin, which is commonly used as a standard for total flavonoids quantification.

The absorbance (A) of each crude extract of *Millettia speciosa* Champ was determined by a UV-Vis spectrophotometer at the maximum absorption wavelength of rutin at 510.0 nm. Subsequently, the mass concentration of total flavonoids in *Millettia speciosa* Champ was calculated according to the standard equation in section 2.3.2. The extraction rate was then determined using equation (1):


$$Rat\;=\frac{(0.0714A+0.0021)\times V\times N\times 10^{-3}}{m}\;\times 100\%$$ (1)

The extraction rate of total flavonoids from *Millettia speciosa* Champ is represented by *Rat* (%). In this equation, *A* denotes the absorbance, *V* represents the volume of the liquid being measured (in mL), *N* indicates the total dilution factor of the test solution, and *m* signifies the mass of *Millettia speciosa* Champ powder (in grams).

### 2.8. Determination of total flavonoids stability

#### 2.8.1. Effect of light.

The diluted crude extract of *Millettia speciosa* Champ was exposed to three different environmental conditions: natural sunlight, indoor conditions, and dark environments. Natural Sunlight: Samples were exposed to natural sunlight for durations of 1, 2, 3, 4, 5, 6, and 7 days. Indoor: Samples were placed on the laboratory bench for the same durations of 1, 2, 3, 4, 5, 6, and 7 days. Dark: Samples were stored in a dark cabinet to prevent light exposure for 1, 2, 3, 4, 5, 6, and 7 days. After the designated exposure periods, the samples were analyzed to evaluate changes in total flavonoids content. The absorbance was measured, and the retention rate was calculated using Equation (2):


$$Ret\;=\frac{Rat_{1}}{Rat_{2}}\;\times 100\%$$ (2)

The retention rate (*Ret*) is defined as the ratio of total flavonoids remaining in the sample (*Rat*_1_) to the initial amount of the total flavonoids present in the sample (*Rat*_2_).

#### 2.8.2. Effect of temperature.

The diluted crude extract of *Millettia speciosa* Champ was placed in a constant temperature water bath at 20, 30, 50, 70, and 90°C. After 30 min, it was removed and cooled to room temperature. The absorbance was measured using a spectrophotometer, and the total flavonoids retention rate was calculated according to Equation (2).

#### 2.8.3. Effect of pH.

The diluted crude extract of *Millettia speciosa* Champ was added to 0.1 mol/L hydrochloric acid and sodium hydroxide solution to adjust the pH to 1, 2, 4, 6, 7, 8, 10, and 12. After fully shaking and standing for 10 min, the absorbance was measured using a spectrophotometer, and the total flavonoids retention rate was calculated according to Equation (2).

#### 2.8.4. Effect of metal ions.

The diluted crude extract of *Millettia speciosa* Champ was added to 0.2 mol/L KCl, NaCl, CaCl_2_, MgCl_2_, CuCl_2_, ZnCl_2_, AlCl_3_ and FeCl_3_ solutions 0 mL, 0.05 mL, 0.10 mL, 0.15 mL, 0.20 mL, 0.25 mL, 0.30 mL, 0.1 mL. After fully shaking and standing for 30 min, the absorbance was measured using a spectrophotometer, and the total flavonoids retention rate was calculated according to Equation (2).

#### 2.8.5. Effect of food additives.

Sodium benzoate was added to the diluted crude extract of *Millettia speciosa* Champ. in mass fractions of 0, 0.03, 0.06, 0.09, 0.12, 0.15, 0.18, 0.21, and 0.24. Additionally, sodium chloride, ascorbic acid, citric acid, glucose, and sucrose were included in mass fractions of 0, 0.1, 0.2, 0.3, 0.4, 0.5, 0.6, and 0.7. The mixture was thoroughly shaken and allowed to stand for 30 minutes. Subsequently, the absorbance was measured using a spectrophotometer, and the total flavonoids retention rate was calculated according to Equation (2) [28].

**Fig 1:**
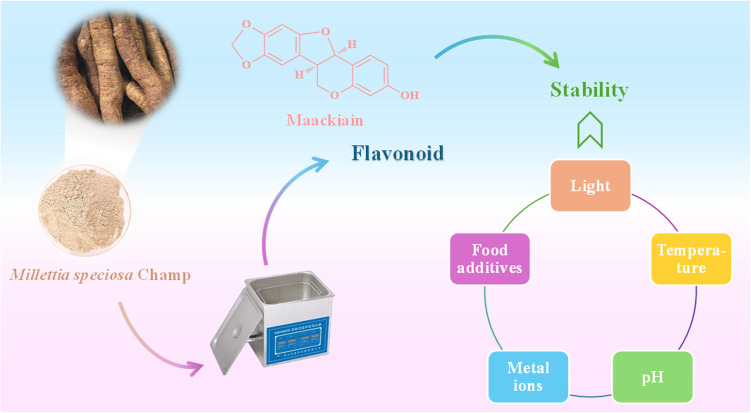
The preparation process of extraction and stability of total flavonoids from *Millettia speciosa* Champ.

The experimental process of UAE and stability was illustrated in Fig 1.

## 3. Results and discussion

### 3.1. Single factor experiments on the extraction of total flavonoids

**Fig 2:**
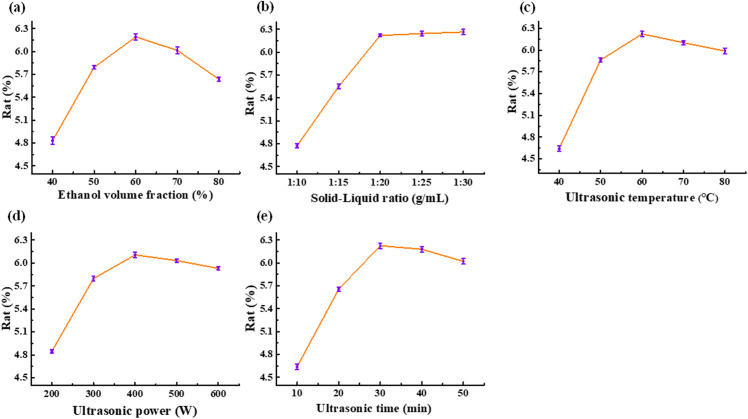
(a) Effect of ethanol volume fraction (b) effect of solid-liquid ratio (c) effect of ultrasonic temperature (d) effect of ultrasonic power (e) effect of ultrasound time on total flavonoid extraction rate from *Millettia speciosa* Champ.

The effect of ethanol volume fraction on the extraction rate of total flavonoids is shown in Fig 2a. With the increase in ethanol volume fraction, the extraction of total flavonoids from *Millettia speciosa* Champ first increased and then decreased, and reached a maximum when the volume fraction was 60%. This may be because with the increase in ethanol volume fraction, the polarity of the extraction solvent is close to the polarity of total flavonoids in *Millette speciosa* Champ, which is beneficial to the dissolution and precipitation of total flavonoids. When the ethanol volume fraction continued to increase, the polarity of the extraction solvent gradually decreased, and the polarity difference between the extraction solvent and total flavonoids in *Millettia speciosa* Champ began to increase, resulting in the obstruction of the extraction of total flavonoids in *Millettia speciosa* Champ [29,30]. Therefore, the 60% ethanol volume fraction was suitable for extracting total flavonoids from *Millettia speciosa* Champ.

The effect of the solid-liquid ratio on the extraction rate of total flavonoids is shown in Fig 2b, with the increase of solid-liquid ratio, the extraction of total flavonoids increased. This may be because an increase in the solid-liquid ratio, can promote solid-liquid contact, thereby reducing the dissolution resistance of total flavonoids [31]. When the solid-liquid ratio was 1:20 g/mL, the solid-liquid ratio continued to increase, the extraction rate of total flavonoids was basically unchanged, and considering the extraction cost, it was more appropriate to choose 1:20 g/mL.

The effect of ultrasonic temperature on the total flavonoid extraction rate is illustrated in Fig 2c. The total flavonoid extraction efficiency initially increased with rising ultrasonic temperature, peaking at 60°C, and then declined. This trend can be attributed to the enhanced solubility of total flavonoids in the extractant at elevated temperatures. However, excessively high temperatures may induce thermal decomposition of certain total flavonoids, structural degradation, and increased dissolution of impurities, ultimately reducing the extraction efficiency [32]. Therefore, an ultrasonic temperature of 60°C was identified as optimal for the extraction of total flavonoids from *Millettia speciosa* Champ.

The effect of ultrasonic power on the total flavonoid extraction rate is presented in Fig 2d. The total flavonoid extraction efficiency initially increased with rising ultrasonic power, reaching a maximum at 400 W, and then declined. This trend can be attributed to the enhanced disruption of cell walls and improved dissolution of total flavonoids at moderate ultrasonic power levels. However, excessive ultrasonic power may lead to the precipitation of other soluble cellular impurities, hindering total flavonoids dissolution. Additionally, higher ultrasonic power generates increased heat, elevating the local extraction temperature and potentially causing thermal degradation of total flavonoids, thereby reducing the extraction efficiency [33]. Consequently, an ultrasonic power of 400 W was determined to be optimal for the extraction of total flavonoids from *Millettia speciosa* Champ.

The effect of ultrasonic time on the total flavonoids extraction rate is illustrated in Fig 2e. The total flavonoids extraction efficiency initially increased with prolonged ultrasonic time, reaching a maximum at 30 min, and then decreased. This trend can be explained by the continuous release of total flavonoids during the initial stages of ultrasonic treatment. However, prolonged ultrasonic exposure may lead to the degradation of thermally unstable total flavonoids components and structural damage due to excessive energy input [34]. Therefore, an ultrasonic treatment time of 30 min was identified as optimal for the extraction of total flavonoids from *Millettia speciosa* Champ.

### 3.2. Optimization of extraction conditions

The experiments were carried out based on a 2.6 factorial design. Experimental data were processed and analysed using SPSS V20 software. The results of the orthogonal experiments, including the analysis of extreme deviations, are presented in S2 Table, while the analysis of variance (ANOVA) is detailed in S3 Table.

**Fig 3:**
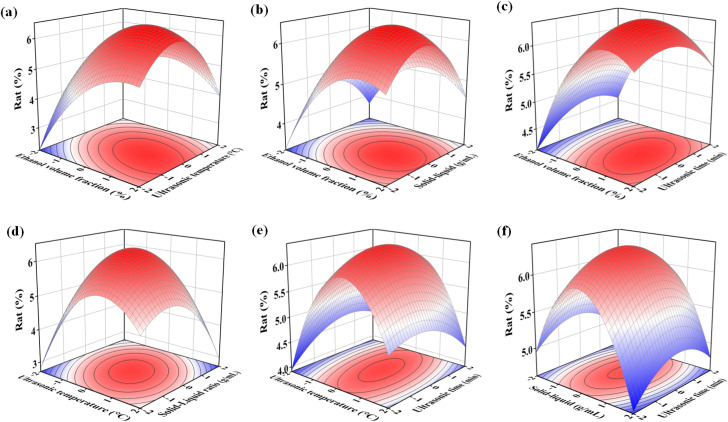
Response surface diagram of interaction, e (a) ethanol volume fraction and ultrasonic temperature, (b) ethanol volume fraction and solid-liquid ratio, (c) ethanol volume fraction and ultrasonic time, (d) ultrasonic temperature and solid-liquid ratio, (e) ultrasonic temperature and ultrasonic time, (f) solid-liquid ratio and ultrasonic time.

According to results and range analysis of orthogonal experimental (S2 Table), and variance analysis of orthogonal experimental results (S3 Table), calibrated model is very significant, among the five influencing factors of ultrasonic-assisted extraction of total flavonoids, the effect of the ethanol volume fraction and the solid-liquid ratio on extraction rate is very significant, the effect of the solid-liquid ratio on extraction rate is significant, and the effect of ultrasonic time on extraction rate is not significant [35]. The effect of various factors from large to small is ethanol volume fraction > solid-liquid ratio > ultrasonic time > ultrasonic temperature > ultrasonic power. The optimal process combination was as follows: A2B2C3D2E2, i.e., the ethanol volume fraction of 60%, the ultrasonic temperature 60°C, the solid-liquid ratio 1:25 g/mL; the ultrasonic time 30 min, and the ultrasonic power 400 W. Based on this, a four-factor quadratic regression orthogonal experiments (fixed ultrasound power 400 W) were designed as shown in S4 Table. The experimental results (Table 1) were processed by SAS R8.01, and the results are analysed in Table 2–4. The response surface and contour diagram are shown in Fig 3.

**Table 1:** Quadratic regression orthogonal experimental results.

Num.	A	B	C	D	Extraction Rate (%)	Num.	A	B	C	D	Extraction Rate (%)
1	−1	−1	−1	−1	4.399	19	0	−2	0	0	4.680
2	−1	−1	−1	1	4.579	20	0	2	0	0	4.768
3	−1	−1	1	−1	4.653	21	0	0	−2	0	5.152
4	−1	−1	1	1	5.129	22	0	0	2	0	5.080
5	−1	1	−1	−1	5.599	23	0	0	0	−2	5.881
6	−1	1	−1	1	5.353	24	0	0	0	2	5.917
7	−1	1	1	−1	5.082	25	0	0	0	0	6.350
8	−1	1	1	1	5.168	26	0	0	0	0	6.230
9	1	−1	−1	−1	5.683	27	0	0	0	0	6.478
10	1	−1	−1	1	5.879	28	0	0	0	0	6.248
11	1	−1	1	−1	5.979	29	0	0	0	0	6.323
12	1	−1	1	1	5.801	30	0	0	0	0	6.432
13	1	1	−1	−1	6.074	31	0	0	0	0	6.372
14	1	1	−1	1	5.922	32	0	0	0	0	6.469
15	1	1	1	−1	5.418	33	0	0	0	0	6.254
16	1	1	1	1	5.249	34	0	0	0	0	6.454
17	−2	0	0	0	4.730	35	0	0	0	0	6.329
18	2	0	0	0	5.958	36	0	0	0	0	6.260

**Table 2:** Variance analysis of the model.

Source	Degrees of freedom	Sum of Squares	Mean Square	F-Value	Pr-Value
A	1	3.0097	3.0097	196.3621	0.0001
B	1	0.1567	0.1567	10.2206	0.0043
C	1	0.0554	0.0554	3.6139	0.0711
D	1	0.0029	0.0029	0.1909	0.6666
A*A	1	1.7589	1.7589	114.7561	0.0001
A*B	1	0.6088	0.6088	39.7192	0.0001
A*C	1	0.0920	0.0920	5.9998	0.0232
A*D	1	0.0399	0.0399	2.6032	0.1216
B*B	1	4.8534	4.8534	316.6518	0.0001
B*C	1	0.5826	0.5826	38.0073	0.0001
B*D	1	0.0834	0.0834	5.4397	0.0297
C*C	1	2.7181	2.7181	177.3393	0.0001
C*D	1	0.0035	0.0035	0.2290	0.6372
D*D	1	0.2931	0.2931	19.1200	0.0003
Model	14	14.2583	1.0185	66.4466	0.0001
Error	21	0.3219	0.0153		
Total	35	14.5802			

**Table 3:** Analysis of variance for regression equations.

Source of variance	Degrees of freedom	Sum of Squares	R^2^	F Value	Pr
Linear	4	3.224681	0.2212	52.60	<0.0001
Quadratic	4	9.623536	0.6600	156.97	<0.0001
Cross product	6	1.410088	0.0967	15.33	<0.0001
Total	14	14.258305	0.9779	66.45	<0.0001

**Table 4:** Lack of fit test analysis.

Source of variance	degrees of freedom	Sum of Squares	Mean Square	F Value	Pr > F
Lack of Fit	10	0.230015	0.023002	2.75	0.0558
Pure Error	11	0.091859	0.008351		
Total Error	21	0.321874	0.015327		

As can be seen from Table 2, the A*B and B*C effects of the primary terms A and B, all the secondary terms, and the interaction terms are highly significant (Pr < 0.01), and the response surfaces in Fig 3a, 3d show highly distorted single peaks. Because the interaction was very significant, the slope and curvature varied greatly in different directions, and the long axes of the ellipses of the contour lines (S2 Fig a, d) were very close to the diagonal, with large variations in the long and short axes, reflecting the fact that the interaction was extremely sensitive to the experimental results.

The effects of interaction terms A*C and B*D were significant (0.01 < Pr < 0.05), and the response surface in Fig 3b,3e showed a distorted monoclinic shape, with the long axis of the ellipse of the contour (S2 Fig b, e) close to the diagonal, and the large variations in the long and short axes reflecting that the interaction was sensitive to the experimental results, hence, During the extraction of total flavonoids from *Millettia speciosa* Champ, The interaction of the factors has a large impact on the experimental results, so the process conditions of the extraction should be strictly controlled, and when one of the factors is changed, the other factors should be considered to be adjusted in order to ensure a higher extraction rate. As can be seen from Table 3, the model is highly significant. Therefore, the results of the experiment can be analyzed by replacing the experimental true points with this regression equation. Table 3 shows that the coefficient of determination R^2^ = 0.9779, i.e., all changes in the independent variables in the regression equation account for 97.79 percent of the variation in the dependent variable. The portion of the total variance that is explained by the model is relatively large, the independent variable explains the dependent variable to a high degree, and the sample observations are close to the regression line. Taken together, these aspects show that the model fits the experimental data well. This is mainly because the influencing factors in the extraction process are not limited to the four variables listed in Table 1 (i.e., the influencing factors), and that the variables that are not accounted for in the equation, as well as the interactions between these variables and the S5 Table variables, affect the regression equation to some extent. On the other hand, the coefficient of determination is not very close to 1 due to the inevitable random errors inherent in experimental procedures. However, only 2.21% of the total variation remains unexplained by the model. As shown in Table 4, the misfit test yields a Pr > 0.05, indicating that the model fits the experimental data well. The misfit term is insignificant compared to the absolute error, suggesting that the influence of misfit factors is negligible. Consequently, the regression model is free from misfit factors and can be effectively used to analyze and predict the total flavonoid extraction rate (S6 Table).

Combining the analyses presented above, it is evident that this regression equation can effectively substitute the experimental data points for predictive analysis of the results. The final regression equation obtained is:


$$\begin{array}{c} Y=6.349917+0.354125*A+0.080792*B-0.048042*C+0.011042*D-0.234448*A*A\\ -0.195063*A*B-0.075812*A*C-0.049938*A*D-0.389448*B*B-0.190813*B*C\\ -0.072188*B*D-0.291448*C*C+0.014813*C*D-0.095698*D*D \end{array}$$

The predicted optimal conditions are as follows: A = 0.409221, B = −0.021638, C = −0.089299, D = −0.076676, with a predicted maximum value of 6.497 occurring at a stationary point.

The predicted optimal values were substituted back into S4 Table, resulting in the optimal process conditions obtained were: the ethanol volume fraction of 64%, the ultrasonic temperature 60°C, the solid-liquid ratio 1:20 g/mL, the ultrasonic time 29 min, the ultrasonic power of 400 W. These conditions yielded an average extraction rate of 6.485% after conducting three parallel experiments.

### 3.3. Determination of total flavonoids stability

#### 3.3.1. Effect of light.

**Fig 4:**
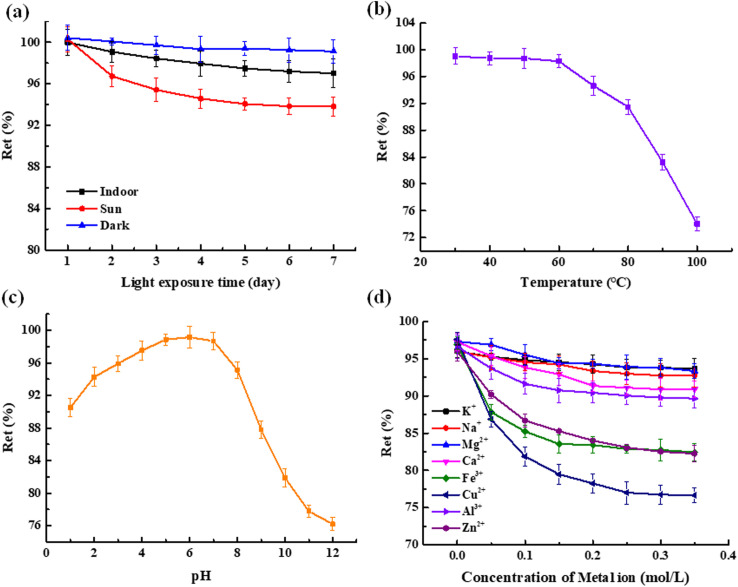
(a) Effects of light (b) effect of temperature (c) effect of pH (d) effect of metal ions of different concentrations on the stability of total flavonoids in *Millettia speciosa* Champ.

The experiment was conducted in accordance with section 2.8.1. The results are presented in Fig 4a. The *Ret* decreased with increasing illumination time. Under sunlight, the rate of decrease of *Ret* was the fastest, under the indoor light source, the rate of decrease of the *Ret* was relatively flat and in the dark environment, the rate of decrease of the *Ret* was the slowest. These may be due to *Millettia speciosa* Champ total flavonoids are easy to decompose under light, and the stronger the light is, the greater the degradation rate [27,36]. Therefore, the extract of total flavonoids from *Millettia speciosa* Champ should be preserved in the dark in actual production.

#### 3.3.2. Effect of temperature.

The experiment was conducted in accordance with section 2.8.2. The results are presented in Fig 4b. With the increase in temperature, the *Ret* gradually decreased, indicating that elevated temperatures compromised the structure of total flavonoids. Therefore, the extract of total flavonoids from *Millettia speciosa* Champ should be preserved at low temperatures (not more than 50°C).

#### 3.3.3. Effect of pH.

The experiment was conducted in accordance with section 2.8.3. The results were presented in Fig 4c. As can be showed that with increasing pH, the *Ret* gradually increases then decreased. The results showed that the extract of total flavonoids from *Millettia speciosa* Champ was stable under neutral and weakly acidic conditions, and unstable under acidic and alkaline conditions. This was derived from total flavonoids have phenolic hydroxyl groups in the structure, which had a certain degree of weak acidity. Under alkaline conditions, the acid-base reaction easily occurred, which had a strong destructive effect on the total flavonoids extract. Therefore, the extract of total flavonoids from *Millettia speciosa* Champ should be preserved under neutral and weakly acidic conditions.

#### 3.3.4. Effect of metal ions.

The experiment was conducted according to 2.8.4. The results were shown in Fig 4d. It can be seen from Fig 4d that metals K^+^, Na^+^, Mg^2+^, Ca^2+^ and Al^3+^ had no significant effect on the stability of total flavonoids extracts from *Millettia speciosa* Champ, while Cu^2+^, Zn^2+^ and Fe^3+^ had a great influence [37]. This was because total flavonoids easily reacted with Cu^2+^, Zn^2+^ and Fe^3+^ to form substances with different colours, resulting in an increase in the *Ret* of the extract. Therefore, the extract of total flavonoids from *Millettia speciosa* Champ should be avoided when using such metal containers in the preservation process.

#### 3.3.5. Effect of food additives.

**Fig 5:**
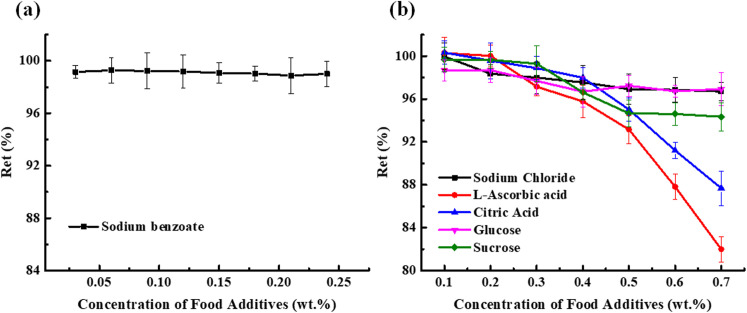
(a) Effect of food additives (Sodium benzoate) on the stability of flavonoids; (b) Effect of food additives (Sodium Chloride, L-Ascorbic acid, Citric Acid, Glucose, Sucrose) on the stability of flavonoids.

The experiment was conducted according to 2.8.5. It can be seen from Fig 5a that in the range of 0–0.25 mass fraction of sodium benzoate, no significant change in *Ret*. As shown in the Fig 5b, in the range of 0–0.7 mass fraction of sodium chloride, ascorbic acid, citric acid, glucose, sucrose, the *Ret* decreased with increasing amounts of ascorbic acid and citric acid, while the increase amounts of sodium chloride, glucose and sucrose reduced the *Ret* but was not obvious. This is because the hydroxyl groups in ascorbic acid, citric acid, glucose, and sucrose molecules are oxidized as hydrogen donors, thus protecting the adjacent hydroxyl groups in the structure of total flavonoids from oxidation to quinone. The above five commonly used food additives have little effect on the total flavonoids at low concentrations. At high concentrations, the pH is small, high acidity, and ascorbic acid and citric acid have a large effect.

## 4. Conclusions

This study focused on optimizing the ultrasound-assisted extraction of total flavonoids from *Millettia speciosa* Champ and assessing the stability of total flavonoids under various environmental conditions. The optimized extraction parameters resulted in a yield of 6.485%. Stability assessments revealed that total flavonoids are best preserved in dark conditions and exhibit stability in neutral and mildly acidic environments, and instability in strongly acidic and alkaline conditions. The presence of certain metal ions, including K^+^, Na^+^, Ca^2+^, Mg^2+^, and Al^3+^, did not significantly affect the stability of the total flavonoids, whereas Cu^2+^, Zn^2+^, and Fe^3+^ were found to diminish stability. Additionally, food additives such as sodium benzoate, sodium chloride, glucose, and sucrose had negligible effects on stability, while ascorbic acid and citric acid significantly compromised stability at elevated concentrations. These results contribute to a more precise assessment of the potential applications of total flavonoids from *Millettia speciosa* Champ as antioxidants and provide a theoretical foundation for their future practical applications.

## Supporting information

S1 Fig Standard curve. (PDF)

S2 Fig Contour plots. (PDF)

S1 Table Orthogonal experimental design. (PDF)

S2 Table Results and range analysis of orthogonal experimental. (PDF)

S3 Table Variance analysis of orthogonal experimental results. Note: R^2^ = 0.996.
(PDF)

S4 Table Response surface design factor level table. Note: The ultrasonic power fixed at 400 W.
(PDF)

S5 Table Variance analysis of factors. (PDF)

S6 Table t test of the model. (PDF)
