# Supplementary figures and images for: Study on the extraction and stability of total flavonoids from Millettia speciosa Champ
Source: PLoS One. 2025 Jul 2;20(7):e0326570. doi: 10.1371/journal.pone.0326570 (PMC12221088; doi:10.1371/journal.pone.0326570)

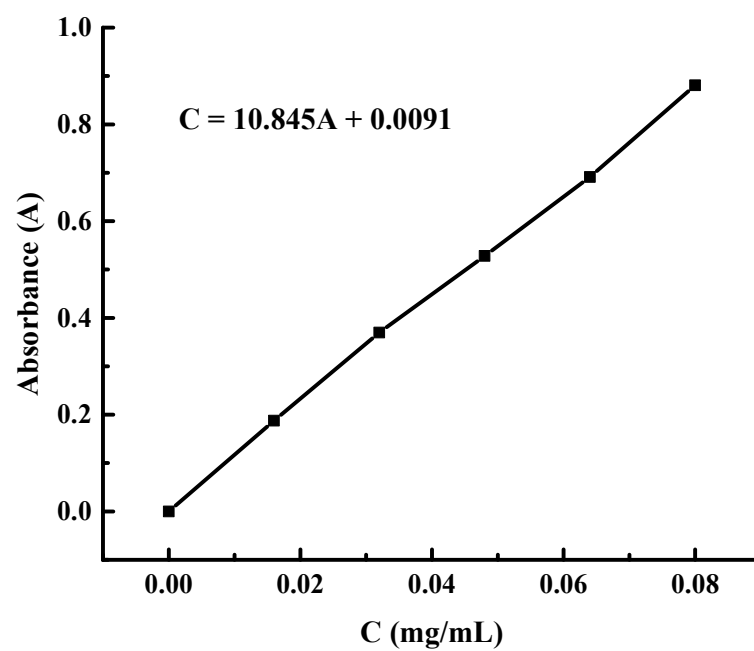

S1 Fig. standard curve.

Supplement: S1 Fig — (PDF) [file pone.0326570.s001.pdf]

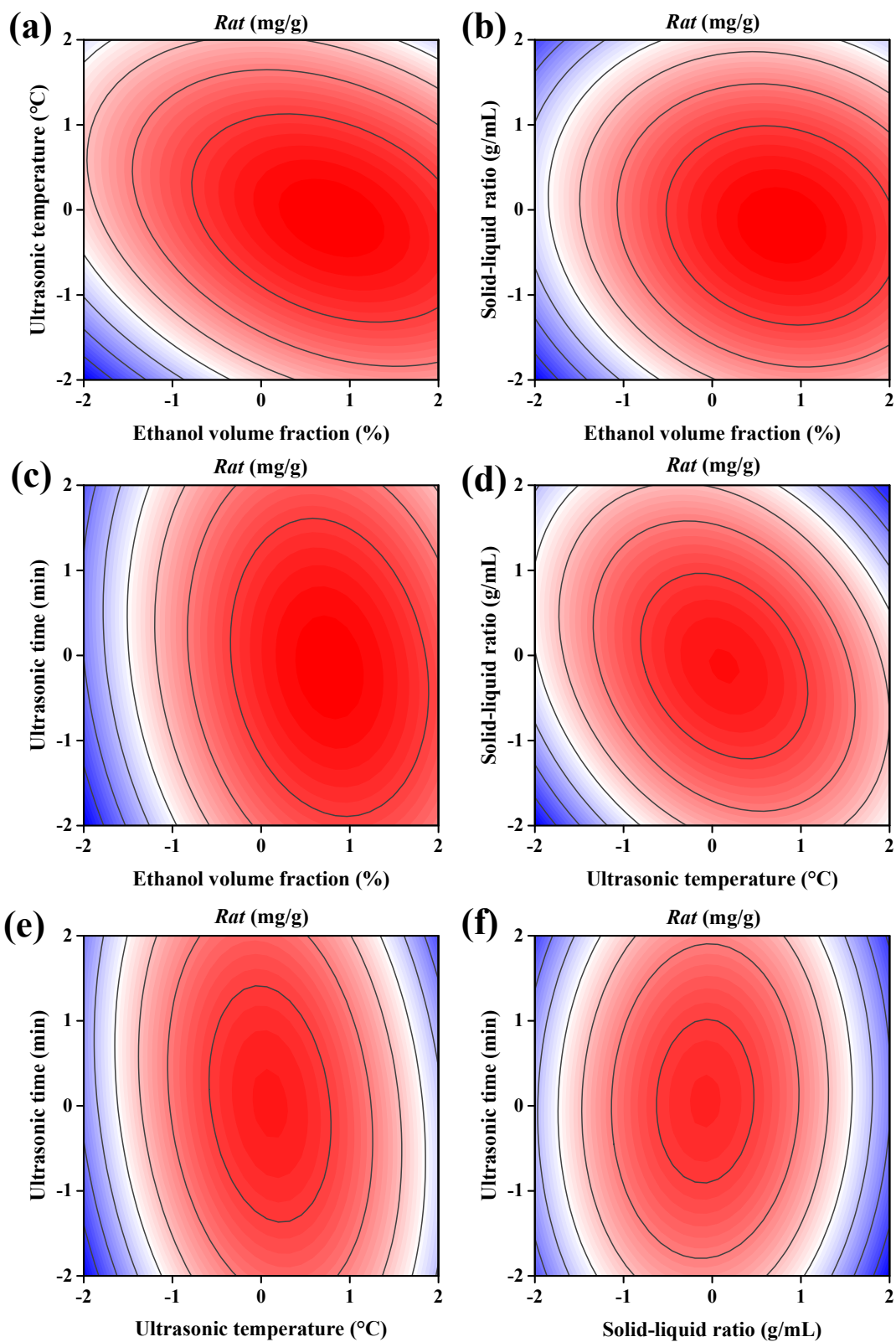

S2 Fig. Contour plots.

Supplement: S2 Fig — (PDF) [file pone.0326570.s002.pdf]
