# Supplementary material for: Study on the extraction and stability of total flavonoids from Millettia speciosa Champ
Source: PLoS One. 2025 Jul 2;20(7):e0326570. doi: 10.1371/journal.pone.0326570 (PMC12221088; doi:10.1371/journal.pone.0326570)
Supplement: S1 Table — (PDF) [file pone.0326570.s003.pdf]

**S1 Table.** Orthogonal experimental design

| Levels | Factors | | | | |
|---|---|---|---|---|---|
| | A | B | C | D | E |
| 1 | 50 | 50 | 1:15 | 20 | 300 |
| 2 | 60 | 60 | 1:20 | 30 | 400 |
| 3 | 70 | 70 | 1:25 | 40 | 500 |
| 4 | 80 | 80 | 1:30 | 50 | 600 |
