# Supplementary material for: Study on the extraction and stability of total flavonoids from Millettia speciosa Champ
Source: PLoS One. 2025 Jul 2;20(7):e0326570. doi: 10.1371/journal.pone.0326570 (PMC12221088; doi:10.1371/journal.pone.0326570)
Supplement: S2 Table — (PDF) [file pone.0326570.s004.pdf]

S2 Table. Results and range analysis of orthogonal experimental

Num.	A	B	C	D	E	Extraction Rate (%)
1	1	1	1	1	1	5.066
2	1	2	2	2	2	5.478
3	1	3	3	3	3	5.407
4	1	4	4	4	4	5.575
5	2	1	2	3	4	5.823
6	2	2	1	4	3	6.144
7	2	3	4	1	2	6.176
8	2	4	3	2	1	6.352
9	3	1	3	4	2	6.417
10	3	2	4	3	1	6.262
11	3	3	1	2	4	5.954
12	3	4	2	1	3	5.761
13	4	1	4	2	3	5.86
14	4	2	3	1	4	5.743
15	4	3	2	4	1	5.422
16	4	4	1	3	2	5.271
K1	5.232	5.642	5.459	5.537	5.626	-
K2	5.974	5.757	5.471	5.761	5.686	-
K3	5.949	5.590	5.830	5.541	5.643	-
K4	5.424	5.590	5.818	5.740	5.624	-
R	0.742	0.167	0.371	0.225	0.062	-
