# Supplementary material for: Study on the extraction and stability of total flavonoids from Millettia speciosa Champ
Source: PLoS One. 2025 Jul 2;20(7):e0326570. doi: 10.1371/journal.pone.0326570 (PMC12221088; doi:10.1371/journal.pone.0326570)
Supplement: S3 Table — Note: R2 = 0.996. (PDF) [file pone.0326570.s005.pdf]

S3 Table. Variance analysis of orthogonal experimental results

Sources of error	Sum of square	Degree of freedom	Mean of square	F-value	Sig.
Calibrated model	2.451	12	0.204	61.899	0.003
Intercept	509.755	1	509.755	154483.829	0.000
A	1.6805	3	0.560	169.715	0.001
B	0.0745	3	0.025	7.517	0.066
C	0.5165	3	0.172	52.170	0.004
D	0.180	3	0.0602	18.194	0.020
Error	0.010	3	0.003	-	-
Total	512.216	16	-	-	-

Note: R^2=0.996
