# Supplementary material for: Study on the extraction and stability of total flavonoids from Millettia speciosa Champ
Source: PLoS One. 2025 Jul 2;20(7):e0326570. doi: 10.1371/journal.pone.0326570 (PMC12221088; doi:10.1371/journal.pone.0326570)
Supplement: S4 Table — Note: The ultrasonic power fixed at 400 W. (PDF) [file pone.0326570.s006.pdf]

S4 Table. Response surface design factor level table

Factors	Level				
	-2	-1	0	1	2
A (ethanol volume fraction, %)	40	50	60	70	80
B (ultrasonic temperature, °C)	40	50	60	70	80
C (solid-liquid ratio, g/mL)	1:10	1:15	1:20	1:30	1:35
D (ultrasonic time, min)	10	20	30	40	50

Note: The ultrasonic power fixed at 400 W
