# Supplementary material for: Study on the extraction and stability of total flavonoids from Millettia speciosa Champ
Source: PLoS One. 2025 Jul 2;20(7):e0326570. doi: 10.1371/journal.pone.0326570 (PMC12221088; doi:10.1371/journal.pone.0326570)
Supplement: S5 Table — (PDF) [file pone.0326570.s007.pdf]

S5 Table. Variance analysis of factors

Factors	Degrees of freedom	Sum of Squares	Mean Square	F Value	Pr > F
A	5	5.509265	1.101853	71.89	<0.0001
B	5	6.284802	1.256960	82.01	<0.0001
C	5	3.451554	0.690311	45.04	<0.0001
D	5	0.422772	0.084554	5.52	0.0021
