# Supplementary material for: Study on the extraction and stability of total flavonoids from Millettia speciosa Champ
Source: PLoS One. 2025 Jul 2;20(7):e0326570. doi: 10.1371/journal.pone.0326570 (PMC12221088; doi:10.1371/journal.pone.0326570)
Supplement: S6 Table — (PDF) [file pone.0326570.s008.pdf]

S6 Table. t test of the model

Term	Estimate	standard error	t-Value	Pr > |t|
Intercept	6.349917	0.035739	177.67	<.0001
A	0.354125	0.025271	14.01	<.0001
B	0.080792	0.025271	3.20	0.0043
C	-0.048042	0.025271	-1.90	0.0711
D	0.011042	0.025271	0.44	0.6666
A*A	-0.234448	0.021886	-10.71	<0.0001
A*B	-0.195062	0.030951	-6.30	<0.0001
A*C	-0.389448	0.021886	-17.79	<0.0001
A*D	-0.075812	0.030951	-2.45	0.0232
B*B	-0.190813	0.030951	-6.17	<0.0001
B*C	-0.291448	0.021886	-13.32	<0.0001
B*D	-0.049938	0.030951	-1.61	0.1216
C*C	-0.072188	0.030951	-2.33	0.0297
C*D	0.014813	0.030951	0.48	0.6372
D*D	-0.095698	0.021886	-4.37	0.0003
